# Old players and new insights: unraveling the role of RNA-binding proteins in brain tumors

**DOI:** 10.7150/thno.113312

**Published:** 2025-04-13

**Authors:** Xu Wang, Jiang Li, Chengkai Zhang, Xiudong Guan, Xingang Li, Wang Jia, Anjing Chen

**Affiliations:** 1 Department of Neurosurgery, Beijing Tiantan Hospital, Capital Medical University, Beijing, China.; 2 China National Clinical Research Center for Neurological Diseases, Fengtai, Beijing, China.; 3 Department of Neurosurgery, Qilu Hospital, Cheeloo College of Medicine and Institute of Brain and Brain-Inspired Science, Shandong University, Jinan, 250012, China.; 4 Jinan Microecological Biomedicine Shandong Laboratory, Jinan, 250117, China and Shandong Key Laboratory of Brain Health and Function Remodeling, Jinan 250012, China.

**Keywords:** RNA-binding proteins, Brain tumors, Glioblastoma, Targeted therapy, Inhibitor.

## Abstract

The human genome harbors >1,600 evolutionarily conserved RNA-binding proteins (RBPs), with extensive multi-omics investigations documenting their pervasive dysregulation in malignancies ranging from glioblastoma to melanoma. These RBPs are integral to the complex regulatory networks governing hallmark cancer processes. Recent studies have investigated the multifaceted contributions of RBPs to tumorigenesis, tumor metabolism, the tumor-immune microenvironment, and resistance to therapy. This complexity is further compounded by the intricate regulation of RNA function at various levels by RBPs, as well as the post-translational modifications of RBPs, which improve their functional capacity. Moreover, numerous RBP-based therapeutics have emerged, each underpinned by distinct molecular mechanisms that extend from genomic analysis to the interference of RBPs' function. This review aims to provide a comprehensive overview of the recent progress in the meticulous roles of RBPs in brain tumors and to explore potential therapeutic interventions targeting these RBPs, complemented by a discussion of innovative techniques emerging in this research field. Advances in deciphering RNA-RBP interactomes and refining targeted therapeutic strategies are revealing the transformative potential of RBP-centric approaches in brain tumor treatment, establishing them as pivotal agents for overcoming current clinical challenges.

## Introduction

Tumorigenesis, a complex and progressive process, poses a significant threat to human health, and brain tumors are among the most formidable types. It starts with the transformation of a benign cell into a precursor cell with tumorigenic potential, driven by genetic mutations that disrupt the balance of pro-oncogenic and anti-oncogenic signaling. In the context of brain tumors, the dysregulation of RNA-binding proteins (RBPs) is a crucial factor contributing to this imbalance 1,2. Current high-throughput screening has effectively discovered more than 1600 RBPs in the human genome, representing around 7.5% of all proteins 3. RBPs play a vital role in maintaining cellular homeostasis and are involved in various stages of the gene expression network 4. They are key regulators of multiple processes in the RNA life cycle, such as splicing, polyadenylation, nucleotide editing, stability, cellular localization, and translation 5. In brain cells, the proper function of RBPs is essential for normalphysiological functions, and any alterations in their expression or function can have a significant impact on the development of brain tumors.

Despite the efforts made in the treatment of brain tumors, the outcomes remain unsatisfactory. Current therapeutic interventions, including surgical resection, radiation, and chemotherapy, have not been able to effectively combat the high incidence and poor prognosis of brain tumors. This highlights the urgent need for new therapeutic strategies. The increasing evidence of the important roles of RBPs in brain tumors, including glioblastoma, meningiomas, medulloblastoma, pituitary adenoma, and brain metastases, offers a new hope 6. Nearly two-thirds of adults with glioblastoma, the most aggressive form of brain tumors, die within two years of diagnosis 7. By modulating RNA-RBP binding, we may be able to target the key molecular mechanisms that drive the growth and progression of brain tumors, and develop more effective and precise therapeutic approaches 8.

The molecular subtyping and gene profiles of brain tumors have provided us with a deeper understanding of their heterogeneity 9. Global changes in post-transcriptional regulation, often mediated by RBPs, are an important contributing factor to the differences in these molecular features among different subtypes of brain tumors 10. RBPs are involved in every step of RNA processing, including splicing, modification, and translation and responsible for the production of more than 74% of human genes in both normal and malignant tissues 11. In the context of brain tumors, the dysregulation of RBPs can lead to significant consequences, making them a highly promising target forprecise treatment.

This review aims to provide a comprehensive overview of the recent advancements in exploring the molecular mechanism of RBPs in brain tumors. It summarizes the potential of RBPs as novel therapeutic targets and the application of RBP-based therapies. By highlighting the importance of RBPs in the development and progression of brain tumors, this review hopes to inspire further research and development in this field, and contribute to the improvement of the treatment outcomes of brain tumors, bringing new hope to patients.

## 1. RBPs' general structure and functional complexes

**Figure 1:**
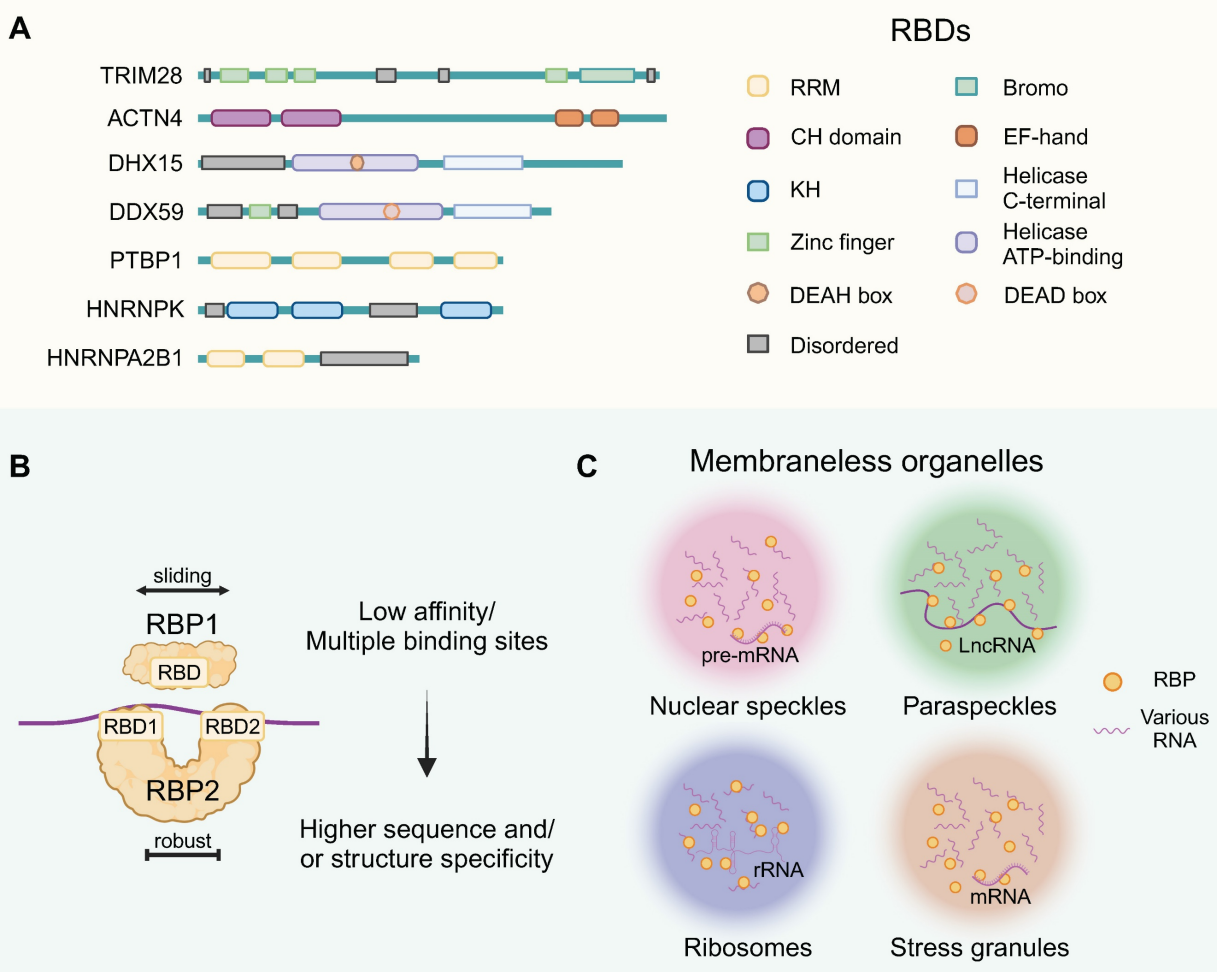
Structural and functional diversity of RBPs in brain tumors. (A) Schematic presentation of the well-defined functional RBDs of RBPs, including domains and motifs. And the intrinsically disordered region was annotated. (B) Protein-RNA Interaction Mechanisms. Individual RNA binding domains typically exhibit lower affinities compared to proteins possessing multiple such domains, with most RBPs featuring multiple RBDs for enhanced specificity in sequence and/or structural recognition. (C) RBP-RNA complexes form MLOs, which are characterized by distinct size, concentration, interactions, and functions. The RNA components within these MLOs include rRNA, snRNA, mRNA, lncRNA, and others. This figure was created with Biorender.

RBPs interact with RNA molecules by recognizing specific sequences or structures through their RNA-binding domain (RBD) configurations. Structurally, these well-defined RBDs include the RNA-recognition motif (RRM), human heterogeneous nuclear ribonucleoprotein (hnRNP) K-homology domain (KH), zinc-finger domains, and the DEAD-box helicase domain, among others (Figure 1A). These abundant mRNA-binding domain classes recognize 4-6 nucleotide segments, usually in combinations or repeats, enhancing RBP binding strength 12. In the meanwhile, nearly half of RBPs lack typical RBDs due to atypical interactions with RNA 13. RBPs are crucial for assembling functional ribonucleoprotein particles (RNPs), primarily through interactions with both coding and noncoding RNAs. The RNP complexes composed of single or tandem RBD-containing proteins bind to their RNA targets, and multiple RBDs could provide higher sequence or structural specificity (Figure 1B).

RNP granules constituted membraneless organelles (MLOs), formed via complex RNA-RNA, RNA-protein, and protein-protein interactions 14. They are notable for their distinct size, concentration and multifaceted interactions (Figure 1C). MLOs can be formed through liquid-liquid phase separation (LLPS) and are thought to be associated with the disordered regions of RBPs in some contexts. This figure highlights the contextual versatility of RBPs, providing a foundation for elucidating the functional mechanisms of RBPs.

Among membraneless organelles, nuclear speckles are found in the nucleus and involved in the storage and assembly of pre-mRNA splicing factors. LLPS of RBP CLK2 initiate the reorganization of nuclear speckles, restricting mRNA splicing and hampering the maintenance of glioblastoma stem cells (GSCs) 15. Paraspeckles contain an architectural lncRNA that attracts proteins to initiate the LLPS process. In GBM, elevated paraspeckle RBP NONO are associated with worse prognosis through abnormal LLPS 16. The ribosome, the cellular machinery for protein synthesis, contains a large amount of rRNA and ribosomal proteins. Based on ribosome-associated RBP signiture, it is possible to establish a prognosis prediction system for GBM 17. Moreover, the absence of RBPs DDX56 and WDR75 disrupts ribosomal protein regulation, inducing nucleolar stress that reduces proliferation and triggers apoptosis in glioma cells through caspase activation 18,19. Stress granules sequester and store untranslated mRNAs during stress conditions, protecting mRNAs from degradation. RBP MSI1 and eIF2a play a leading role in stress granule formation that grants GSC with stemness properties and chemoresistance 20. These are several examples of the oncogenic functions of RBPs within cellular organelles. These highlight the contextual versatility of RBPs, providing a foundation for elucidating the functional mechanisms of RBPs.

## 2. RBPs achieve their function via gene expression regulation

**Figure 2:**
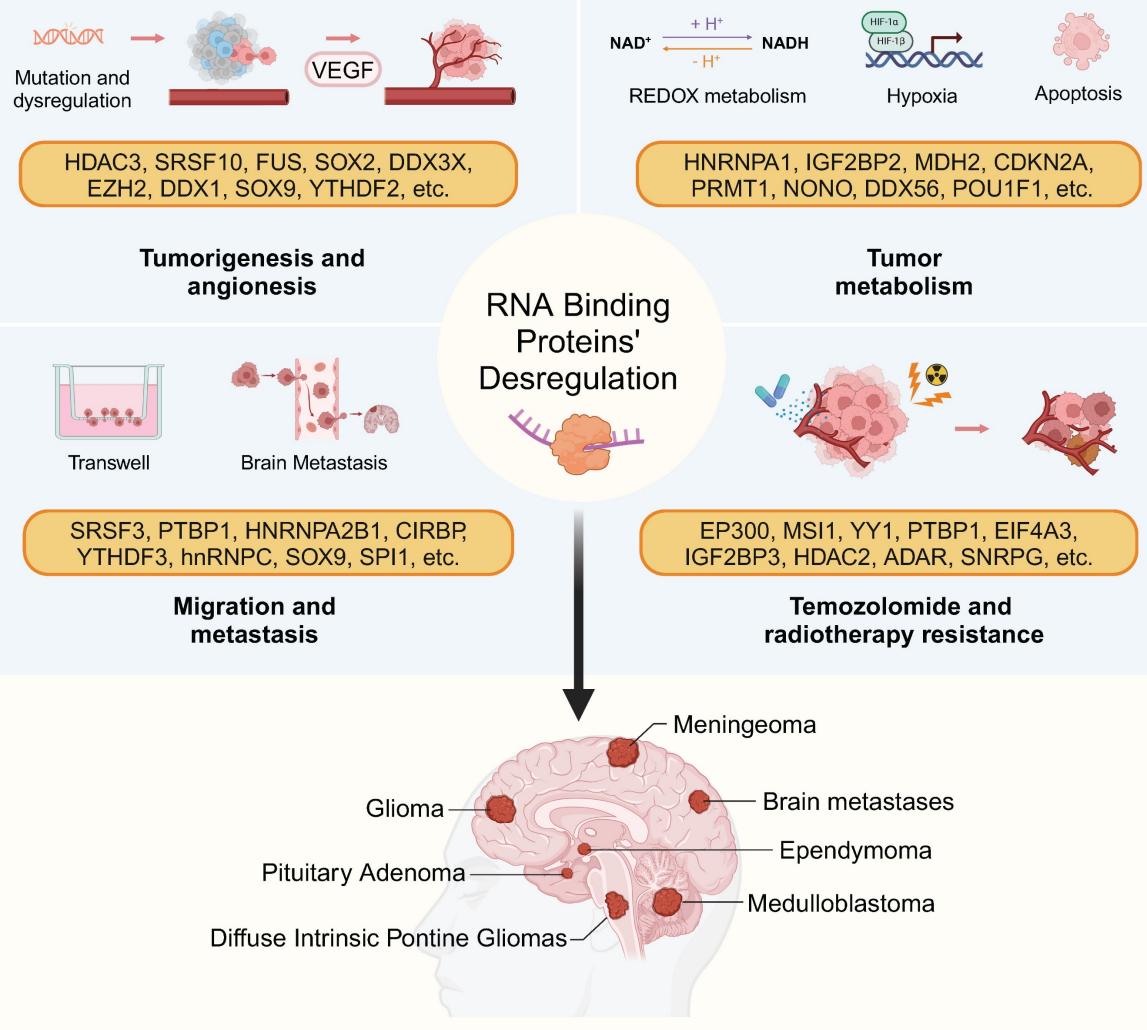
RBP-driven hallmarks of brain tumor pathogenesis. RBPs govern tumor angiogenesis, metabolic reprogramming, therapy resistance, and immune evasion through context-dependent RNA regulation. This figure was created with Biorender.

This part outlines the complex interactions of RBPs with regulatory noncoding RNAs (ncRNAs) and mRNA in cancer biology, emphasizing RBP roles in gene expression and biological function. It's clear that RBP-RNA complexes are diverse in brain tumors and forming regulatory networks. The relationship between RBP dysregulation and tumor behavior is summarized in Figure 2.

### 2.1 RBPs in epigenetic regulation

Epigenetic changes result in inheritable gene expression alterations without DNA sequence alteration. In brain tumors, RBPs can exert epigenetic regulation on their downstream target genes.

Mechanistic studies of the long noncoding RNA (lncRNA) HOTAIR have shown that its 5' region binds PRC2 for H3K27 trimethylation, while the 3' region binds LSD1/CoREST/REST for H3K4 demethylation 21. HOTAIR modulates cell cycle progression in GBM via its interaction through RBP EZH2 and PRC2 complex 22. Histone H2B acetylation is partially driven by RBP EP300 in classical-like and immune-low GBM, as determined by stratification based on snRNA-seq 23. Histone deacetylases (HDACs) play significant roles in gliomas by regulating gene expression, with their inhibitors showing potential therapeutic value. Lnc00461 stability is maintained through interactions with HDAC6 and RBPs like CNOT6 and FUS. Targeting Lnc00461 in GBM can decrease cell division-related proteins and subsequently induces cell cycle arrest 24. RBP HDAC2 promotes the binding of c-Myc to the CCL1 promoter, thereby activating tumor immunosuppression in GBM 25. Diminished RBP PRMT1 and H4R3me2 levels are observed in IDH1R132H gliomas. The PRMT1-PTX3 axis is essential for controlling ferritin genes/iron storage, and its inhibition triggers ferritinophagic flux 26.

### 2.2 RBPs in transcription

RBPs also regulate tumorigenesis through transcription independently of histone modifications. RBP Ddx3x governs hindbrain development and prevents medulloblastoma by modulating Hox genes. It senses oncogenic stress in mice prone to Wnt- or Shh-driven medulloblastoma, suppressing tumorigenesis 27. RBP SOX9, together with FOXG1, reshapes the enhancer activation landscape, driving EGFRvIII-dependent tumorigenesis in GBM 28. Furthermore, RBP UPF1 binds to circRPPH1, maintaining its stability. This interaction forms a feedback loop with transcription factor ATF3, contributing to a stable stream that enhances tumorigenesis in GSCs 29. p53 maintains quiescence by promoting fatty acid oxidation in neural stem cells (NSCs) and suppressing tumor initiation. The upregulation of transcriptional induction of RBP PPARGC1a activates PPARα, leading to the upregulation of fatty acid oxidation genes 30. Previous research has pinpointed multiple transcription factors involved in pituitary development and tumor formation. Both POU1F1 (also known as PIT-1) involved in somatotroph, lactotroph, and thyrotroph adenoma, and NR5A1 (also known as SF-1) involved in gonadotroph adenoma, are RNA-binding proteins 31,32.

RBPs influence tumor metabolism and invasion through transcriptional regulation. Hypoxia, characteristic of glioblastoma microenvironment, elevates miR-1246 in glioma-derived exosomes through increased transcription and selective packaging, assisted by RBPs such as POU5F1 and hnRNPA1 33. Moreover, the hypoxia-inducible lncRNA HIF1A-AS2, specific to the mesenchymal subtype, is upregulated in GSCs. It interacts with RBPs IGF2BP2 and DHX9 to sustain HMGA1 expression 34. The RBP SPI1 plays a complex role in transcriptional modulation during GBM progression. SPI1 induced MIR222HG expression in MES-GBM tissues and inhibited the transcription of FTO in GBM 35,36. RBP Pax6 severely impairs tumor propagation by downregulating Olig2 in PDGF-induced oligodendroglioma and is proposed as a positive prognostic marker for gliomas 37,38.

### 2.3 RBPs in pre-mRNA splicing and processing

RBPs play a crucial role in RNA splicing by modulating spliceosome assembly and splice site selection. Glioblastomas can be distinguished from healthy brain samples by the altered expression of key spliceosome components and splicing factors, including SRSF family, PTBP1 39. Overexpression of circSMARCA5 boosts the inclusion of exon 4 in the SRSF3 RNA isoform, typically skipped by SRSF1, creating an nonsense-mediated decay (NMD) substrate that promotes glioma cell migration 40. The lncRNA LINREP regulates PTBP1-mediated AS, notably skipping RTN4 exon 3 to produce diverse splice variants 41. The splicing of a brain-enriched cassette exon in the tumor suppressor ANXA7 is mediated by PTBP1, which subsequently enhances EGFR signaling during glioma progression 42.

In another research, 58 consistently upregulated RBPs in GSCs were identified by measuring the expression levels of 1542 human RBPs in GBM and glioma stem cell samples 43. The EGFRvIII upregulates the splicing factor hnRNPA1 through mTOR and MYC, leading to hnRNPA1-dependent splicing of the protein Max. This results in the promotion of glycolytic gene expression and significantly shorter survival in glioma patients 44. The splicing factor hnRNPH regulates the aberrant splice event IG20, creating an anti-apoptotic isoform that redirects TNF-α/TRAIL-induced death signals to promote survival 45. RBM47 promotes the inclusion of exon 20 of TJP1, and the resulting TJP1 isoform improves the assembly of actin stress fibers, thereby promoting EMT in neuroblastoma 46. The RBP DHX15 acts as a tumor suppressor gene in glioma, suppressing the expression of NF-κB downstream target genes involved in splicing 47. In summary, the splicing variants mediated by RBPs in brain tumors are highly diverse and still requeired further exploration.

In addition to splicing, RBPs directly bind to RNA to regulate RNA processing. RBP QKI has been identified as the primary regulator of the biogenesis of circular RNAs during EMT, indicating its involvement in promoting brain tumor invasion 48. SRSF10 enhances circ-ATXN1 production in glioma by binding its 5' and 3' ends. Circ-ATXN1, within an RNA-induced silencing complex, sequesters miR-526b-3p to modulate glioma angiogenesis 49. EIF4A3-induced circRNA ASAP1 expression, along with flanking regions, promotes tumorigenesis and temozolomide resistance in glioblastoma 50. Additionally, eIF4A3 induced cyclization of circMMP9, resulting in increased circMMP9 expression in GBM by binding to the MMP9 mRNA transcript 51. The m6A modification on pri-miR-10a is recognized by HNRNPA2B1, which then recruits processor DGCR8 to facilitate pri-miR-10a processing 36.

### 2.4 RBPs in mRNA modification

N6-methyladenosine (m6A), the most prevalent mRNA modification, is abundant in coding sequences and 3'-UTRs, especially in long internal exons and near stop codons. m6A proteins--methyltransferases (METTL3/METTL14), demethylases (FTO/ALKBH5), and binding proteins--play key roles in mRNA processing, particularly in stability and translation regulation 52. Human antigen R (HuR) recruitment was essential for the stabilization of lncRNA LINREP via m6A formation, which implicated in the regulation of PTBP1-induced AS 53. YTHDF2 enhances UBXN1 mRNA decay through METTL3-catalyzed m6A methylation, which boosts NF-κB activity. Elevated UBXN1 levels counteract the tumorigenic impact of YTHDF2 overexpression and correlate with improved GBM patient survival 54.

As for other types of RNA modifications, 5-Methylcytosine (m5C) occurs not only in tRNAs and rRNAs but also in mRNA 55. NSUN5, which introduces m5C at the C3782 site in human 28S rRNA, induces a general reduction in protein synthesis, facilitating an adaptive translational response for glioma cell survival under stress 56. TET proteins (TET1/2/3) catalyze the oxidative conversion of DNA and RNA m5C to hm5C and f5C, respectively, and dysregulation of TET family proteins has been implicated in glioma 57. As for N1-methyladenosine (m1A) modification and pseudouridine (ψ) modification, the m1A eraser ALKBH1 and pseudouridine synthases (PUSs, ψ writers) also function as RBPs and play crucial roles in GBM 58,59. The upregulation of NAT10, an enzyme responsible for N4-acetylcytidine (ac4C) modification, promotes the acetylation of PARP1, thereby inducing cell death in GBM cells 60. CFIm25 modulates proximal poly(A) site selection through alternative polyadenylation (APA). Knockdown of CFIm25 results in the shortening of 3'UTRs in over 1,400 genes, subsequently leading to elevated expression of oncogenes in GBM 61. However, these mRNA modifications in brain tumors still require further research.

### 2.5 RBP in translational and post-translational regulation

The translation process is facilitated by RBPs from the Superfamily II DNA and RNA helicase family within the ribosome, and the efficiency of translation is closely related to mRNA stability. The lncRNA ATXN8OS stabilizes GLS2 mRNA by recruiting RBP ADAR, thereby limiting TMZ resistance in glioma 62. Additionally, CSTF2 interacts with the mRNA of the BAD to truncate its 3'-UTR, adversely affecting BAD-mediated apoptosis and promoting the survival of GBM cells 63. YBX2 binds and stabilizes HNF4G mRNA to regulate blood-tumor barrier (BTB) permeability 64. There is a potential mechanism of APA in GBM involving the influence of multiple RBPs on the stability and translation of APA isoforms 65. It is noteworthy that there are also examples of RBPs negatively regulating RNA stability. The RBP HNRNPD can decrease the stability of ZHX2 mRNA and promote the formation of vasculogenic mimicry in GBM 66.

Elevated YTHDF3 RBP levels facilitats blood-brain barrier (BBB) penetration and angiogenesis. YTHDF3 boosts translation of m6A-rich ST6GALNAC5, GJA1, and EGFR transcripts, which are involved in brain metastasis 67. The Msi1 mRNA has an extensive 3'-UTR that contains several AU- and U-rich sequences, often targeted by HuR. The HuR binds to the Msi1 3'-UTR, improving MSI1 mRNA stability and translation in GBM 68. RBP YBX1 binds to the 5'-UTR of CCT4 mRNA to promote the translation of CCT4, which activates the mTOR signaling pathway 69. Previous research has shown that global eIF4E-mediated translation is inhibited in glioma 70. However, stable silencing of DDX28 in hypoxic human glioblastoma cells increased eIF4E2 binding to the m7GTP cap structure and translation of eIF4E2 target mRNAs 71.

**Figure 3:**
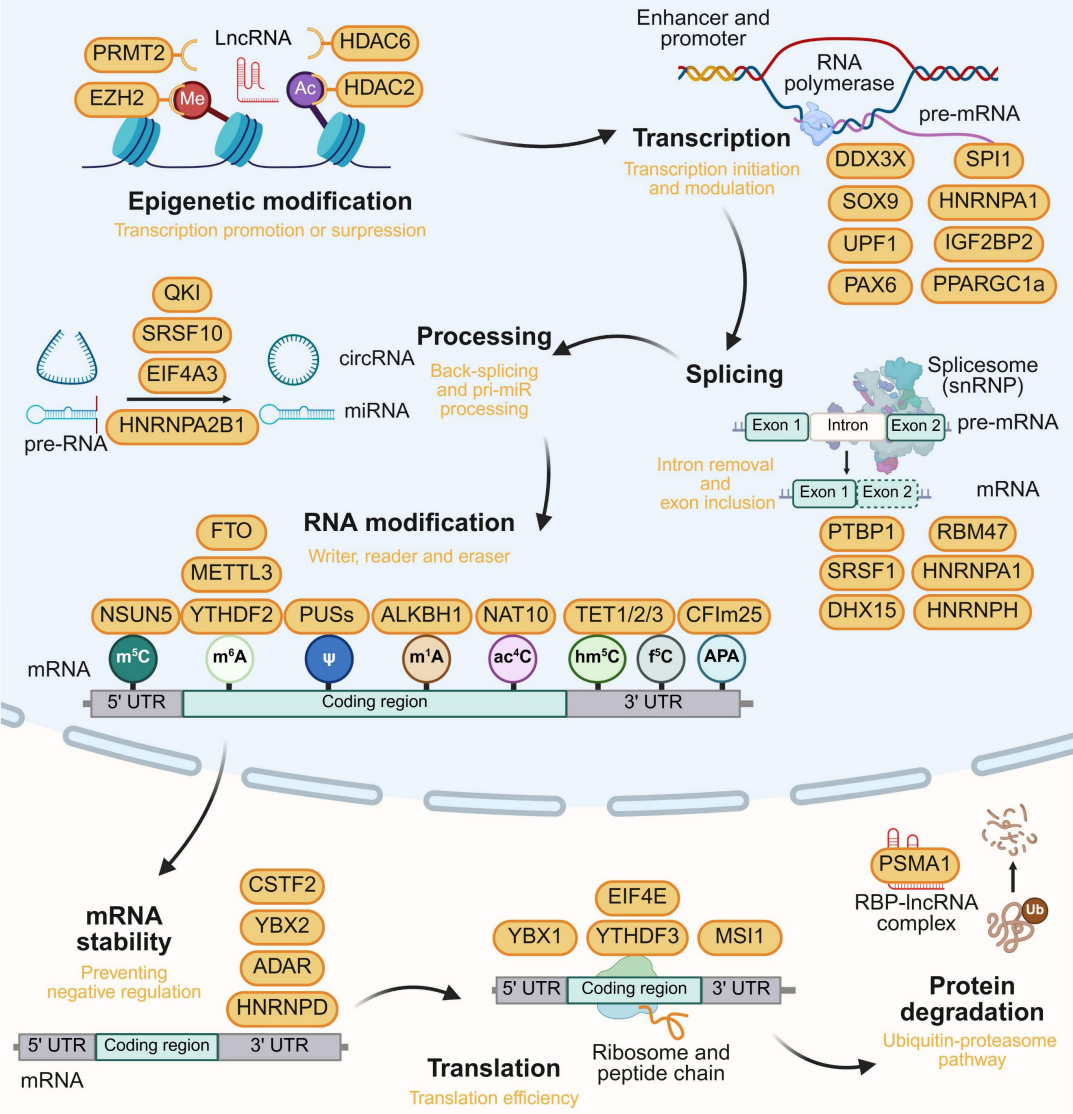
Multi-layered regulatory roles of RBPs in gene expression. RBPs modulate epigenetic modification, transcription, splicing, RNA processing RNA modification, mRNA stability, translation and protein degradation, with representative oncogenic/tumor-suppressive RBPs annotated at each regulatory layer. This figure was created with Biorender.

In post-translational regulation, suppression of RBP SNRPG facilitates the cytosolic translocation of MYC and nuclear translocation of p53. This enhances the inhibitory effect of TMZ and reduces the mismatch repair protein MLH1 in GBM 72. LncRNA MDHDH serves as a molecular scaffold, binding RBP PSMA1 and MDH2, to promote MDH2 degradation, affecting mitochondrial membrane potential and the NAD^+^/NADH balance, ultimately impeding GBM glycolysis 73. The role of RBPs in brain tumors is listed in Table 1 and the model is summarized in Figure 3. This hierarchy emphasizes the potential of stage-specific RBP targeting to disrupt tumorigenic networks.

## 3. RBPs' expression and function regulation

### 3.1 RBPs' expression regulation

This complex interaction network between RBP and ncRNA has been gradually revealed in the context of brain tumors. The SNP rs72780850 variant in the RBP DDX1 promoter shows enhanced transcription factor binding and is linked to increased DDX1 expression, potentially elevating neuroblastoma risk 74. Continuous radiation stimulates the nuclear translocation of p65. Consequently, there is increased expression of RBP YY1, which induces radioresistance in glioma cells 75. CDKN2A expression, a RBP biomarker for aggressive meningiomas, is regulated by genetic aberrations and miRNAs in meningiomas and pilocytic astrocytomas 76,77.

The lncRNA HOXA-AS3 functions as a mir-455-5p sponge, upregulating RBP USP3 expression to facilitate GBM progression 78. PTB-AS significantly increases PTBP1 level by directly binding to its 3'-UTR, and masking the miR-9 binding site in the PTBP1-3'-UTR to prevent PTBP1 degradation 79. lncRNA NEAT1 indirectly regulates RBP SOX2 expression by targeting miR-132 in glioma 80. miR-146a diminishes the expression of RBP hnRNPC, which then inhibits the migratory and invasive activity of brain metastatic cells by downregulating the expression of MMP-1, uPA, and uPAR 81. LINC00470 lncRNA dynamically regulates RBP subcellular localization by forming a ternary complex with FUS and AKT in the cytoplasm, which activates AKT, prevents nuclear translocation, and fosters glioblastoma tumorigenesis 82.

### 3.2 RBPs' function regulation by protein modification

Protein modifications play a significant role in the regulation of brain tumors by RBPs, as previously reported. The PPARGC1A K224R mutant expression significantly increased mitochondrial biogenesis under hypoxic conditions. This effect is attributed to the loss of PPARGC1A K224 monomethylation, which is regulated by oxygen availability-dependent KDM3A 83. HDAC3 modulates the acetylation state of STAT3 and competes with STAT3 for P300 binding, thereby antagonizing astrogliogenesis. This regulation influences phenotypic commitment during oligodendrocyte-astrocytic fate decision 84. Mutant p53 increases ZDHHC5 and NF-Y expression, enhancing GSCs via EZH2 palmitoylation and phosphorylation modifications 85.

The cyclic adenosine 3′,5′-monophosphate pathway activation promoted RBP FLNA phosphorylation in GH-secreting tumoral pituitary cells, abolishing antitumor effects on pituitary cells 86. Cerebellar granule cell precursor proliferation is governed by Hedgehog signaling. AMP-activated protein kinase phosphorylates RBP CNBP at threonine 173 upon Hedgehog signaling, enhancing medulloblastoma cell proliferation 87. Additionally, DNA-PKcs phosphorylates RBP SOX2 at S251, stabilizing SOX2 by preventing WWP2-mediated ubiquitination. Consequently, this stabilization promotes the maintenance of GSCs 88.

RBP HNRNPL prevents ACTN4 from being degraded by the proteasome, a process involving ubiquitination. This protection enhances NF-κB subunit's nuclear accumulation and contributs to GBM progression 89. Moreover, LINC00998 stabilizes RBP CBX3, prevents its ubiquitination degradation, and regulates the c-Met/Akt/mTOR signaling pathway in GBM 90. RBP DDX31 binds to NPM, blocking the interaction between E3 ubiquitin ligase and p53, leading to p53 stabilization in medulloblastoma tumorigenesis 91. Increased levels of RBP SOX9 correlate with medulloblastoma spread and unfavorable outcomes. The phosphorylation of SOX9 at T236 competes with its ubiquitination by E3 ligase FBW7 92.

**Figure 4:**
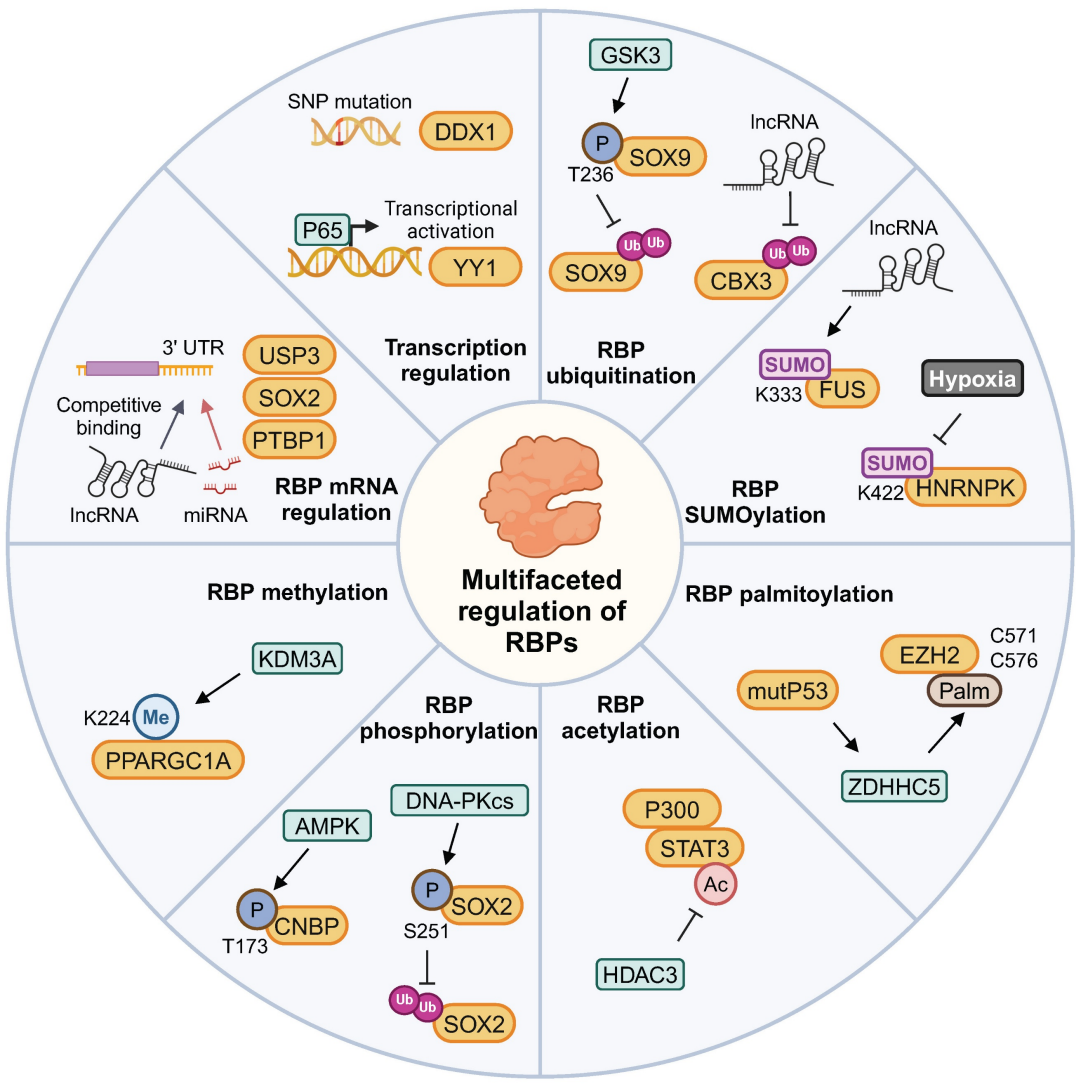
The regulation of gene expression and post-translational modifications of RBPs in brain tumors. The post-translational modifications of RBPs include methylation, phosphorylation, acetylation, palmitoylation, ubiquitination, and SUMOylation. This figure was created with Biorender.

SUMO (Small Ubiquitin-like Modifier) is a small ubiquitin-like protein that participates in post-translational modification of proteins, with particularly high levels of SUMOylated proteins observed in GBM. Hypoxia enhances the cytoplasmic translocation of hnRNPA2B1 by enhancing its SUMOylation, a process that can be inhibited by the SUMOylation inhibitor TAK-981 93. The lncRNA RMST enhances RBP FUS SUMOylation, particularly promoting SUMO1 attachment at K333. This modification strengthens FUS-hnRNPD interaction, stabilizing these proteins and inducing mitophagy 94. GSCs preferentially exhibit high levels of SUMO1-modified SUMOylation. The SUMO1 SUMOylation inhibitor juglone significantly abrogated GSC maintenance and GSC-driven tumor growth 95. The expression of HNRNPK-SUMO1 is primarily localized within the infiltration regions of GBM. The SUMOylation of the K422 residue on HNRNPK impairs its capacity to bind DNA, consequently disrupting subsequent transcriptional processes 96. The multifaceted regulatory patterns of RBPs are summarized in Figure 4. Focusing on these modifications provides a strategic approach to indirectly modulate RBP function within the context of tumor biochemistry.

## 4. RBP influences on brain tumor microenvironment

### 4.1 RBP functions in cellular microenvironment

**Figure 5:**
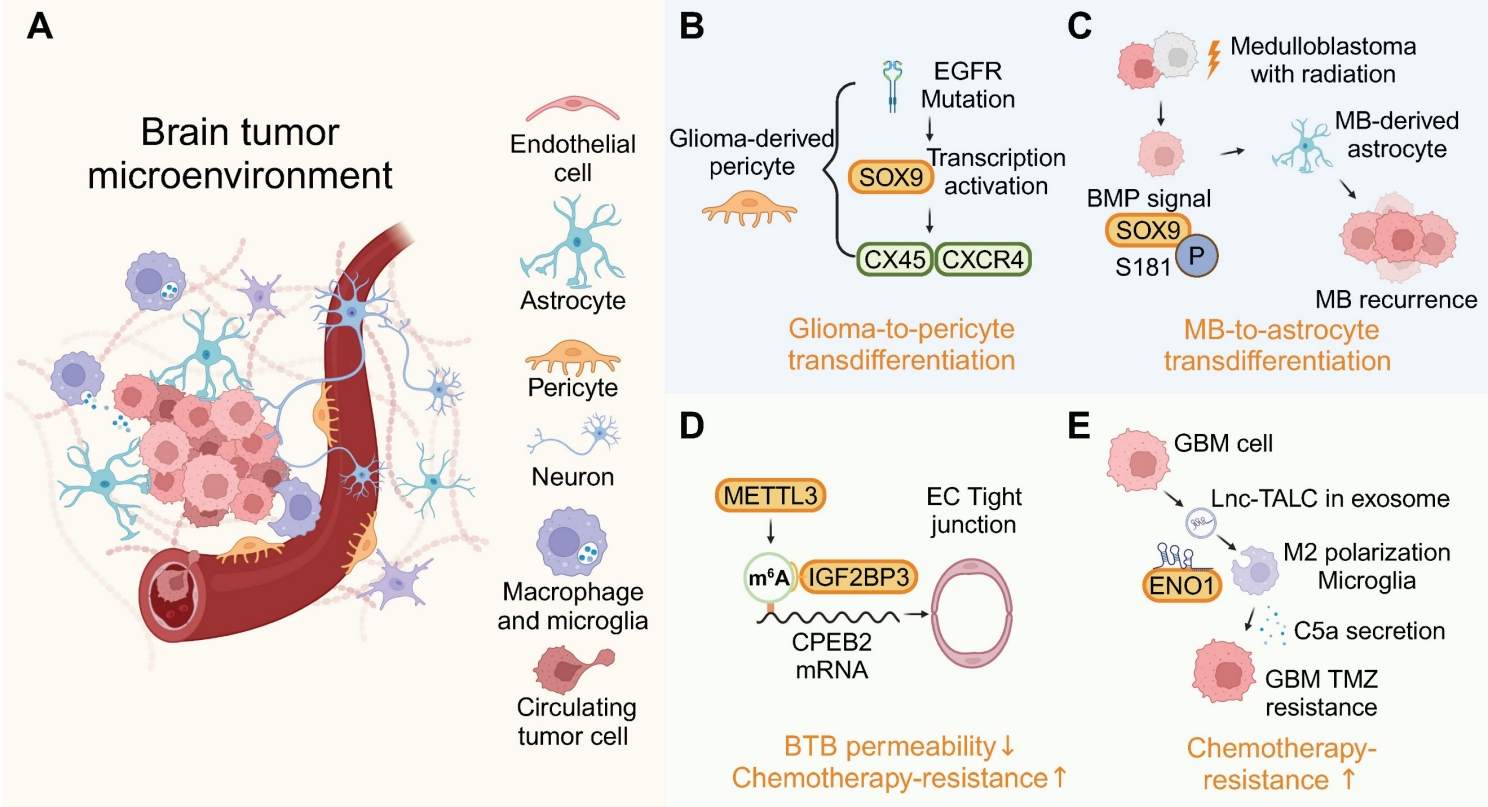
RBP-mediated crosstalk in the tumor microenvironment. (A) RBPs drive cellular plasticity in endothelial cells, astrocytes, and immune cells to foster a pro-tumorigenic niche. (B-E) Mechanistic case studies illustrate RBP roles in transdifferentiation, and chemotherapy resistance. This figure was created with Biorender.

While the BBB is widely recognized as a major obstacle in the treatment of brain tumors, the role of RBPs within the BBB remains poorly understood. RBPs are involved in BBB-mediated brain tumor progression through various cellular microenvironments (Figure 5A).

Dysregulation of RBPs can notably affect cell phenotypes in the brain's microenvironment. EGFR mutations prompt glioma cells to act as pericytes, dependent on BMX/SOX9, thereby stabilizing blood vessels and attracting immune cells via a glioma-to-pericyte transition 97 (Figure 5B). Astrocytes are derived from the transdifferentiation of tumor cells in relapsed medulloblastoma. Treatment with BMP proteins significantly induced astrocytogenesis and phosphorylation of the RBP SOX9, effectively stimulating tumor cell transdifferentiation into astrocytes (Figure 5C) 98. The accumulation of microglia in the brain tumor microenvironment is associated with malignancy and a poor prognosis for these tumors. Activation of TLR2 along with other TLRs transforms microglia into a glioma-supportive phenotype 99. RNP complex LOC-DHX15 promote infiltration of immunosuppressive glioma-associated microglia and macrophages 100.

TARBP2 is an RBP bound to SNHG7, leading to an extended half-life of SNHG7 and, consequently, an increased permeability of BBB 101. Upregulation of RBP METTL3 and IGF2BP3 in glioma microvascular endothelial cells (ECs) increased the stability of CPEB2 mRNA through m6A methylation, thereby regulating BTB permeability (Figure 5D) 102. The expression of RBP YBX2 could influence BTB permeability through YBX2-mediated transcriptional activities 64. Recent GBM research underscores the reciprocal signaling between tumors and neurons, perpetuating a cycle of increased proliferation, synaptic incorporation, and heightened brain activity 103. Furthermore, RBP nucleolin plays a crucial role in the axonal trafficking of mRNA and influences neuronal growth, highlighting the multifaceted functions of RBPs in both the tumor and its microenvironment 104. By analyzing patient samples and patient-derived xenograft (PDX) models that recapitulate the invasive pattern, it is suggested that overexpressed RBP CIRBP is essential for brain metastasis 105.

### 4.2 RBP functions in extracellular vesicles

Extracellular vesicles (EVs), including exosomes, are cellular messengers that transport various molecules including RNA. Recent studies have revealed that RBPs are present within EVs, with some RBPs playing a role in the selective packaging of ncRNAs into exosomes. RBP hnRNPA2B1, MSI, AGO2, and YBX1 have been implicated in the selective sorting of specific RNAs into exosomes 106. Moreover, the RBP-RNA interaction was primarily detected through their binding site in EVs. For example, RBPs could be detected in EVs through inferred RNA targets, or EV-transcripts were found to harbor sequence motifs mirroring the activity of RBPs 107.

RNA within EVs has multiple roles, and the RBP QKI, found in EVs, is linked to primary brain tumors and breast cancer tissues. Elevated QKI levels are associated with poorer survival, indicating its potential as a prognostic marker for glioblastoma and brain metastasis 108,109. Furthermore, RBP-ncRNA-exosome mechanisms are involved in establishing an immunosuppressive microenvironment in brain tumors. circNEIL3, encapsulated by RBP hnRNPA2B1 into exosomes, is transferred to tumor-associated macrophages, conferring them immunosuppressive capabilities via IGF2BP3 stabilization 110. The binding of miR-30b-3p to hnRNPA2B1 facilitates its transfer into EVs. The EV-packaged miR-30b-3p decreased apoptosis and increased the proliferation of glioma 111. RBP hnRNPA1 facilitates the inclusion of exo-miR-1246, which drives monocyte polarization towards myeloid-derived suppressor cells (MDSCs). These MDSCs inhibit CD8^+^ T cell proliferation, aiding in the creation of an immunosuppressive microenvironment 33. As a result of lnc-TALC binding to RBP ENO1, Lnc-TALC could be incorporated into exosomes and transmitted to microglia and promote M2 polarization (Figure 5E) 112. These findings all indicate that the role of EV-associated RBPs in brain tumors can no longer be ignored. The above insights highlight the potential of microenvironment-focused RBP therapeutics as a complementary approach to traditional tumor cell-targeted strategies.

## 5. Targeting RBPs in therapeutic strategy

### 5.1 Engineered particles

Various approaches, such as small-molecule inhibitors, peptides and nanomedicines, are employed to modulate RBPs for therapeutic purposes in cancer treatment, aiming to control gene expression and biological functions.

Engineered particles were designed to target tumor-associated RBPs. Considering the existence of the BBB, corresponding delivery strategies have also been developed for nanomedicines 113. A β-cyclodextrin nanoparticle formulation encapsulating the RBP TLR7/8 agonist R848 was developed to target blood-borne macrophages, bypassing the need for adaptive immunity in the glioma microenvironment 114. Immunotherapy using a TLR9 agonist CpG ODN offers a novel strategy. CpG is efficiently loaded into apolipoprotein E peptide-directed polymersomes, forming a CpG nano-immunoadjuvant that can cross the BBB and target glioma and cervical lymph nodes 115. The RBP WT1-mediated upregulation of CD97 promotes cellular invasiveness 116. Based on this target, a modified 9-mer WT1 peptide was tested in a Phase II clinical trial, indicating the potential application of RBP-related vaccines in GBM 117. The Iodine-123 Meitner-Auger PARP1 inhibitor (123I-MAPi), an iodine-123 labeled PARP1 inhibitor, is a targeted therapy for GBM that utilizes Auger electrons for precise apoptosis induction 118.

### 5.2 Small molecule inhibitors

Developing RBP-targeted therapies involves screening RNAi oligos or small molecules that decrease RBP-RNA binding and modify target RNA levels. Using high-throughput methods to identify RBP-bound RNAs and analyzing large datasets could lead to new cancer treatments or prognostic markers 119.

RBP HDAC2 affects several physiological processes through the deacetylation of histones and non-histones 120. PCI-24781, identified through the Connectivity Map bioinformatic tool, is a novel HDAC2 inhibitor specific to the GBM signature. It works synergistically with TMZ to improve its efficacy and GBM survival 121. The EP300 inhibitor CPI-1612 suppressed H3K27ac and the associated transcription complex and synergistically sensitized TMZ *in vitro* by brain penetration 122. The epigenetic modifier KDM1A inhibitor NCD38 can cross the BBB, and KDM1A inhibition increases the efficacy of TMZ for GBM 123. The palmitoylation of OCT4 is indispensable for protecting it from lysosomal degradation and maintaining GSCs. Its competitive palmitoylation inhibitor, CPP-S1, inhibits the self-renewal ability and tumorigenicity of GSCs 124.

STAT3 is traditionally regarded as a transcription factor. However, it is now widely accepted STAT3 is also a highly versatile RBP 125. The continuous activation of STAT3 in a significant proportion of GBM, which positions STAT3 as a promising therapeutic target and a prognostic marker for GBM 126. The compound SS-4, developed through computational modeling for STAT3 binding, specifically targets STAT3 Y-705 phosphorylation in GBM 127. Treatment with WP1066, a STAT3 inhibitor currently used in clinical settings for pediatric brain tumors, inhibits tumor growth in H3K27M-mutant diffuse midline gliomas 128 and high-grade gliomas with arginine or valine substitutions in the histone H3.3 glycine 34 residue 129. In the case of HuR, the focus is primarily on three ongoing targeted strategies. These include inhibiting HuR translocation from the nucleus to the cytoplasm, inhibiting the ability of HuR to bind to target RNA, and silencing HuR expression. The corresponding inhibitor, MS-444, was verified in GBM 130.

Auranofin was used to disrupt the binding and splicing of the RBP NONO, thereby inhibiting the malignancy of GBM 131. The RBP CDK9 inhibitors, including SNS032, LY2857785, AZD4573, NVP229, and JSH15030, significantly reduced the viability and self-renewal of GBM cells 132. The sensitivity of PLX-4720 is associated with the expression of RBP SPI1 and improves the radiosensitivity of mesenchymal GSCs 35. Inhibition of the RBP SPI1 using DB2313 restored downstream FTO expression and alleviated the tumor burden in GBM 36. Treatment with DHODH inhibitors like brequinar or ML390 depletes pyrimidine levels in glioblastoma and causes nucleolar stress. This stress causes mislocalization of the RBP NPM1 and promotes p53 transcription factor stabilization 133. The PARP-1 inhibitor PJ-34 suppresses the migration of brain microvascular endothelial cells, thereby influencing the growth and progression of brain tumors 134. The selective nanobody NB237 against RBP TRIM28 inhibits the invasiveness and spread of GSCs *in vivo* 135.

METTL3 facilitates medulloblastoma growth through the enhancement of Sonic Hedgehog signaling, and its inhibition by STM2457 impedes tumor advancement 136. The LIN28B inhibitor 1632 reduced medulloblastoma cell growth and provided preliminary preclinical results for drugs for medulloblastoma 137. HB007, identified through a cancer cell-based drug screen, selectively degraded SUMO1 in GBM PDX model 138. Compound 25, discovered via SPR screening, potently binds hnRNPK and disrupts its interaction with the c-myc promoter, offering a selective approach to modulate oncogene transcription without targeting DNA secondary structures 139. Furthermore, TERT and LRP1, both recognized as RBPs, have small-molecule drugs that have progressed to clinical trial stages 140,141.

Finally, considering that most RBPs are difficult to target with drugs, RNA-PROTACs represent a novel approach for targeting RBPs. These chimeric structures employ small RNA mimics as targeting groups that dock the RNA-binding site of the RBP, whereupon a conjugated E3-recruiting peptide directs the RBP for proteasomal degradation 142. This targeting approach has been proven to be effective against the glioma oncogene LIN28.

### 5.3 Antisense oligonucleotides (ASO)

ASOs are short, single-stranded nucleic acids (DNA or RNA) ranging from 12 to 24 nucleotides in length. They are designed to bind to specific complementary mRNA targets through base pairing, thereby modulating gene expression. ASO are categorized into RNase H/L-dependent ASOs that facilitate RNA cleavage and steric blockade ASOs that hinder RBP binding 143. Modifications enhance ASO stability, cellular uptake, and bioavailability for diverse applications 144.

An ASO targeting LINC02283 and the concomitant RBP PDGFRA successfully inhibited GBM tumorigenesis 145. The selected ASOs specifically delay the growth of patient-derived H3.3K27M cells grown by mediating the depletion of the H3-3A protein in diffuse intrinsic pontine gliomas (DIPG) 146.

**Figure 6:**
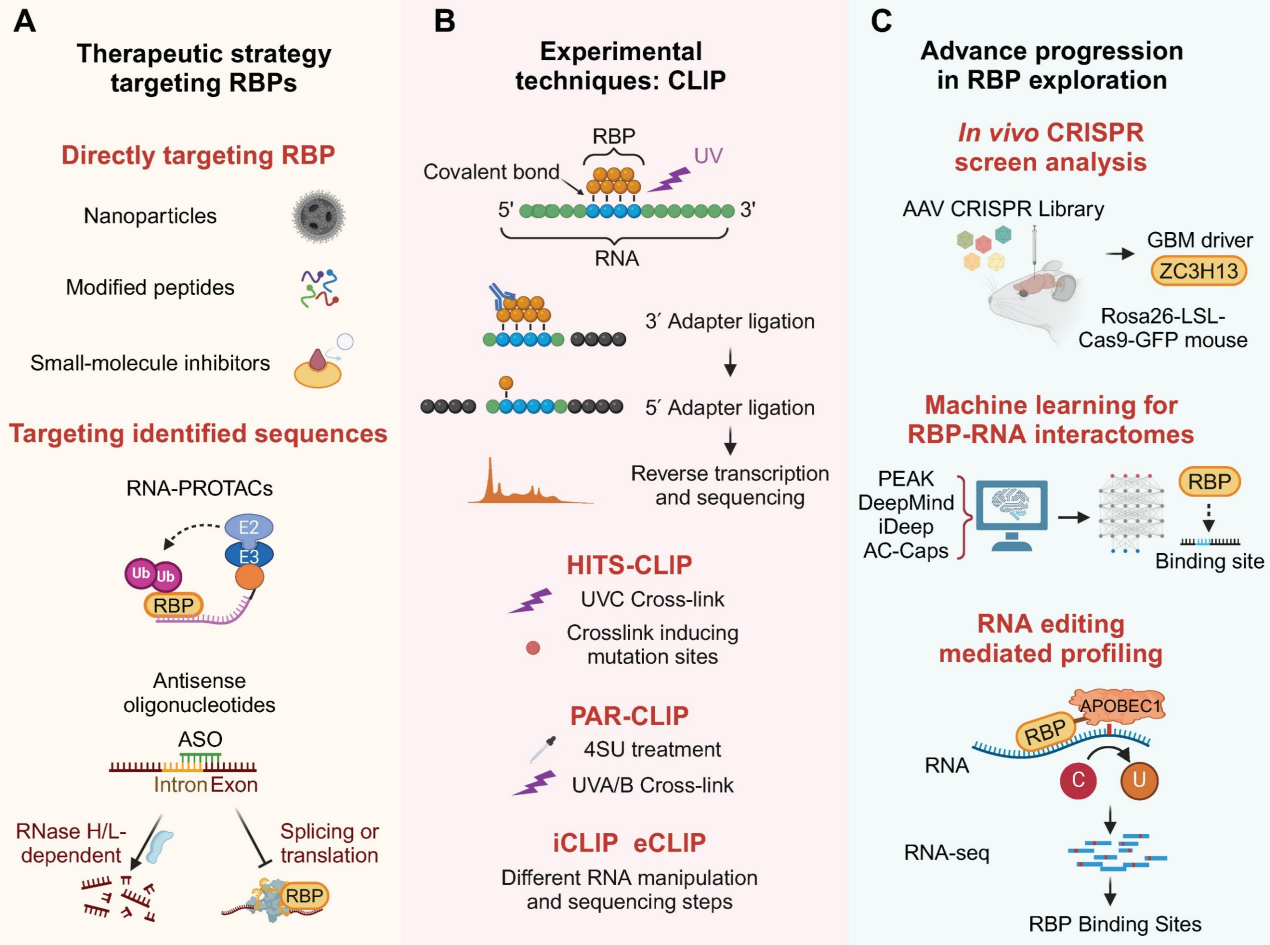
Technological advances in RBP research. (A) Therapeutic modalities targeting RBPs, including nanoparticles, peptides, inhibitors, PROTACs, and ASOs. (B) Evolution of CLIP-based methods for high-resolution RBP-RNA interactome mapping. (C) CRISPR screening and machine learning enable systematic discovery of clinically actionable RBP targets. This figure was created with Biorender.

In addition, a decoy oligonucleotide similar to ASO, composed of several repeats of an RNA motif, can specifically bind to splicing factors such as SRSF1. This binding inhibits their splicing activity and suppresses the growth of gliomas 147. This type of RBP decoy also hinders the translation of mRNA mediated by YBX1 69. And an Aptamer-RIBOTAC designed based on ASO can activate endogenous RNase L to precisely degrade oncogenic miRNAs in gliomas, offering another promising therapeutic approach 148. The strategies targeting RBPs have been summarized in Figure 6A. This panel resonates with the emphasis on innovation in precision medicine, highlighting tools that bridge the gap between mechanistic discovery and clinical translation. The various approaches for targeting RBPs and their specific applications are comprehensively summarized in Table 2.

## 6. Advancements and progressions in RBP networks

### 6.1 Advancements in technologies for RBP-RNA interaction

The information regarding which RNA sequence or structure is bound by the RBPs is crucial. Over the past decades, various methods have been developed to determine this, including RNA immunoprecipitation (RIP) 149, targets of RNA-binding proteins identified by editing (TRIBE) 150, and RNA tagging 151. These techniques have significantly improved our understanding of RNA-protein interactions and their roles in cellular processes. Each method has its strengths and limitations, and the choice of method often depends on the specific research question and available resources.

Crosslinking and immunoprecipitation (CLIP) is a widely used technique for studying protein-RNA interactions *in vivo* on a transcriptome-wide scale, and its applications have expanded over time (Figure 6B). CLIP utilizes ultraviolet (UV) light to create covalent bonds between RNA and attached proteins, followed by immunoprecipitation with specific RBP antibodies to isolate RBP-RNA complexes, initially used to map direct interactions in the brain's RNA network 152.

CLIP's evolution has spawned variants that boost efficiency across steps like RNA fragmentation and RBP purification. HITS-CLIP, which integrates high-throughput sequencing, offers a comprehensive view of RBP-RNA interactions at nucleotide resolution, enabling the study of RNA-binding sites in distinct brain regions 153. Individual-nucleotide CLIP (iCLIP) is an advanced protocol that uses leftover amino acids post-crosslinking to stop reverse transcriptase, allowing for precise mapping of RBP-RNA interactions in GBM. It incorporates a second adaptor with a unique sequencing barcode during circularization for detailed interaction site localization 154. Enhanced CLIP (eCLIP) refines the iCLIP process by incorporating a size-matched control during RNA-protein purification, facilitating a more accurate assessment of the specificity in RNA-PTBP1/IGF2BP2 interactions with HOTAIRM1 155. Photoactivatable ribonucleotide-enhanced CLIP (PAR-CLIP) enhances the mapping of RBP-RNA interactions by incorporating 4-thiouridine or 6-thioguanosine into nascent RNAs, followed by UV-A light-induced crosslinking to RBPs, facilitating precise interaction site identification through RNA-seq analysis in brain. The integration of RNA-Seq data with the application of PAR-CLIP for YBX1 revealed direct post-transcriptional control in medulloblastomas 156.

Nevertheless, the CLIP technique remains dependent on substantial sample sizes and highly efficient RBP antibodies, and it is limited to the analysis of a single RBP at a time. More powerful and precise methods for exploring RBPs are continuously being updated, although they have not yet been applied in the field of brain tumors. A novel approach combining quantitative proteomics with photoreactive nucleotide-enhanced UV crosslinking and oligo (dT) purification has been established to identify mRNA-bound proteins 157. The RNA interactome capture (RIC) technique employs magnetic beads to enrich and analyze the mRNA interactome, thereby overcoming the limitation of relying on highly efficient antibodies 158,159. "cat RNA-Protein Interaction Design" utilizes RNA-binding motifs to forecast protein-RNA interaction probabilities. Its sequence fragmentation method facilitates the analysis of both long linear and circular RNAs 160. LACE-seq, designed to reveal RBP-regulated RNA networks in single oocytes, surpasses various CLIP-based methods in sensitivity, accuracy, precision, and efficiency, potentially offering a clearer view of brain tumorigenesis and the microenvironment at the single-cell level 161. Beyond genomic and transcriptomic analyses, protein-protein interaction networks and dynamic subcellular localization provide a holistic view of the RBPs' mutational landscapes 162,163. It is anticipated that these methods will also be applied to the exploration of brain tumors.

### 6.2 CRISPR screening

CRISPR-Cas9 technology has emerged as a new tool for RBP screening and regulation. Advancements in delivery systems for CRISPR-Cas9 have been crucial for RBP-focused cancer therapies, facilitating the discovery and targeting of cancer-related RBPs. Effective delivery of CRISPR-Cas9 is key to deepening our understanding of RBPs in cancer and their translation into clinical therapeutics 164. Adeno-associated virus-based CRISPR screening in glioblastoma has been used to create tumor models that mimic human disease. This approach involves injecting a library of viruses targeting common cancer genes into the brains of mice with conditional Cas9, leading to the formation of tumors. This method has identified co-occurring driver combinations like RBP Zc3h13-Rb1 and provided insights into the functional suppressors of gliomagenesis. The CRISPR-Cas9 system's efficient delivery is crucial for understanding and advancing RBPs as therapeutic targets in cancer 165.

### 6.3 Multimodal integration and machine learning

With the growing volume of RBP data, it's essential to understand how motif enrichment influences RBP-RNA binding profiles in cells. The ENCODE consortium has generated extensive CLIP data using the eCLIP method on 150 RBPs, providing a rich resource for this research 166. New computational methods such as PEKA have been developed to identify motifs in CLIP data, enabling comprehensive analysis of RBP binding preferences 167. The refinement of CLIP methodologies has significantly accelerated the identification of RBP-RNA interactomes.

The HARD-AP method retrieves RBPs and tightly associated RNA regulatory complexes, and systematically maps RNA-binding sites across primary organs using machine learning-based modeling 168. In addition, other notable deep learning approaches for predicting RBP binding sites include DeepMind and iDeep 169,170. An attention-based capsule network (AC-Caps method) can reliably process large-scale RBP binding site data on lncRNA 171. This integration has, in turn, enabled the construction of predictive models for regulatory networks involved in RBP-RNA interactions within brain.

### 6.4 RNA editing applications

Comprehending RBP functions requires identifying their *in vivo* targets, yet CLIP and its adaptations may face challenges like a lack of suitable antibodies or inefficiency in small cell populations. Several recently designed genetic approaches were developed to identify RBP targets under such circumstances.

TRIBE and similar editing-based profiling techniques are innovative genetic tools for investigating post-transcriptional regulation and RBP detection 172. A novel approach involves modifying sequences near methylation sites, which could enable m6A detection by standard RNA-seq, overcoming limitations of current antibody-dependent methods. The RBP APOBEC1, a cytidine deaminase that targets both DNA and RNA, induces cytidine to uridine (C to U) editing 173. Originally identified for editing ApoB mRNA, APOBEC1 has been repurposed in CRISPR/Cas9 systems for inducing C to U transitions at specific single-stranded DNA locations 174. Fusing APOBEC1 to the m6A-binding YTH domain could enable targeted editing of cytidines near m6A sites in RNAs. Detecting these edits with RNA-Seq may provide a clearer picture of the m6A landscape in brain tumors 67,175. This innovative approach provides a promising avenue for advancing our understanding of the complex dynamics of protein-RNA interactions and their implications in brain tumor pathology. These advanced methods for exploring RBP networks are summarized in Figure 6C.

## 7. Conclusions and perspectives

The past two decades have witnessed a surge in mechanistic studies that have significantly expanded our understanding of RBPs within the RNA regulation network. In addition to their conventional intracellular locations, RBPs can localize to the cell membrane surface, where they facilitate the cell-penetrating peptide entry and engage in communication within the cellular microenvironment 176. Moreover, RBPs not only facilitate the transdifferentiation of cells within the brain microenvironment, as previously mentioned, but also serve as independent prognostic risk factors for brain tumors. They have the potential to predict patient outcomes due to their association with immune checkpoint responses and chemotherapeutic efficacy 177,178. The role of the IGFBP family is of particular interest, as it significantly contributes to the interferon pathway and the activation of T cells and NK cells in various brain tumor types 179,180. These findings indicate that the functions of RBPs in brain tumors are not yet fully understood and continue to possess significant potential value.

With the advent of novel methodologies, a genome-wide perspective on the RNA structure-dependent interactions of RBPs under physiological and pathological states is now possible. This method not only improves our knowledge but also facilitates the discovery of RBP-RNA interactions with therapeutic potential. The field of compound screening is rapidly advancing, with innovative techniques being developed to modulate RBP interactomes, thereby offering new therapeutic avenues. Despite this progress, targeting RBPs presents several challenges, particularly regarding delivery methods and translation of the RBP-RNA network's potential in clinical applications for brain tumor treatment.

The involvement of RBPs in brain tumor pathogenesis has sparked interest in their modulation as a novel therapeutic strategy. Approaches targeting the RNA-RBP network include RNA-based therapies, such as ASOs, siRNAs, RNA aptamers, and mRNAs, which are now part of clinical medicine. Moreover, small molecules that target RNA structures are under development, using strategies like conjugating RNA recognition elements to ribonuclease-activating compounds 181. Compound screening is crucial for identifying protein-based inhibitors that can block RNA-protein interactions, with several small molecules already identified that bind to RBPs and inactivate specific RBDs 182. Additionally, compounds that disrupt the nucleocytoplasmic shuttling of RBPs, such as the YBX1 inhibitor HSc025, offer alternative strategies for modulating RBP functions 183.

The intracellular delivery of therapeutic RNA without inducing immune responses is essential for effective RNA-based RBP therapy. The delivery systems are categorized into polymer-based, lipid-based, and conjugate-based systems, with the ability to target specific cells either passively or actively 184. Active targeting uses specific ligands to bind molecular targets, such as the clinically established GalNAc-linked siRNA and ASO conjugates 185. Biomimetic nanovesicles, including endogenous EVs and artificial nanovesicles, can cross the BBB, making them ideal for brain-targeted drug delivery 186. EVs can serve as delivery platforms for RNAs like miRNAs and siRNAs in cancer therapy 187. The secretory autophagy pathway facilitates the extracellular release of RBPs and RNA cargo via EVs using the LC3-conjugation machinery 188. Exosome targeting can be improved by adding peptides to their surface and loading ncRNAs for therapeutic efficacy, effectively combining targeting and therapeutic capabilities. Overall, for clinical applications of exosome-based therapies, the production and quality control of exosomes must be meticulously addressed 186.

The RNA-RBP regulatory network has emerged as a pivotal axis for advancing brain tumor therapy. Integrating RBP profiles into molecular diagnostics could enable personalized therapies by defining actionable targets across tumor subtypes. Clinically, RBP-targeting strategies (e.g., ASOs and PROTACs) show potential to reprogram oncogenic RNA networks with high specificity. However, challenges remain in delivering these agents across the blood-brain barrier and addressing tumor heterogeneity. Advances in nanovesicles and exosome-based delivery systems may overcome these limitations, while liquid biopsies monitoring exosomal RBPs could refine treatment response assessment 189. Moving forward, efforts should focus on multi-omics mapping of RBP interactions, validating biomarkers in clinical cohorts, and combining RBP inhibitors with immunotherapy or radiation to counter resistance. By aligning mechanistic insights with scalable therapeutic platforms, RBP research embodies the theranostic vision of bridging molecular discovery to patient care.

## Figures and Tables

**Table 1:** The role of RNA binding protein in brain tumor.

	RBP	Cancer role	Cancer type	Cellular mechanisms	Ref.
Histone modification	EZH2	Proto-oncogenic	Glioblastoma	Promote H3K27 trimethylation	22
	EP300	Proto-oncogenic	Ependymoma	Promote H2B acetylation	23
	HDAC2	Proto-oncogenic	Glioblastoma	Deacetylation of histones and non-histones	25,120
	PRMT1	Proto-oncogenic	Glioma	Promote H4R3me2 of PTX3 promoter	26
Transcriptional regulation	DDX3X	Tumor-suppressing	Medulloblastoma	Modulating Hox expression and suppresses medulloblastoma formation	27
	PPARGC1a	Proto-oncogenic	Glioblastoma	Activates PPARα expression in GSCs	30
	POU1F1 and NR5A1	Proto-oncogenic	Pituitary adenoma	Pituitary development and tumorigenesis through transcription	31,32
	IGF2BP2 and DHX9	Proto-oncogenic	Glioblastoma	Sustain HMGA1 expression in mesenchymal subtype GBM	34
	Pax6	Tumor-suppressing	Oligodendroglioma and glioma	Surpressing Olig2 expression	37,38
Splicing and processing	SRSF1	Tumor-suppressing	Glioma	Inclusion of exon 4 in the SRSF3	40
	PTBP1	Proto-oncogenic	Glioblastoma	Involved in exon skipping of RTN4 and ANXA7	41,42
	hnRNPA1	Proto-oncogenic	Glioma	Splicing of the Max mRNA	44
	RBM47	Proto-oncogenic	Neuroblastoma	Promoted the inclusion of exon 20 of TJP1	46
	DHX15	Tumor-suppressing	Glioma	Alterantive splicing of glioma	47
	SRSF10	Proto-oncogenic	Glioma	Enhances circ-ATXN1 production by binding its 5' and 3' ends	49
	EIF4A3	Proto-oncogenic	Glioblastoma	Promoted circular RNA biogenesis and cyclization	50,51
RNA modification	YTHDF2	Proto-oncogenic	Glioblastoma	Enhances UBXN1 mRNA decay through m6A methylation	54
	NSUN5	Proto-oncogenic	Glioma	Introduces m5C at the C3782 site in 28S rRNA	56
	NAT10	Proto-oncogenic	Glioblastoma	Promotes the acetylation of PARP1 mRNA	60
	CFIm25	Proto-oncogenic	Glioblastoma	Introduces APA in mRNA 3'UTRs	61
Translational and post-translational regulation	ADAR	Tumor-suppressing	Glioma	Stabilizes GLS2 mRNA with lncRNA ATXN8OS	62
	HNRNPD	Proto-oncogenic	Glioblastoma	Decrease the stability of ZHX2 mRNA	66
	YTHDF3	Proto-oncogenic	Brain metastasis	Enhances translation of m6A-enriched transcripts	67
	YBX1	Proto-oncogenic	Glioblastoma	Bounds the 5′UTR of CCT4 to promote the translation	69
	EIF4E2	Proto-oncogenic	Glioblastoma	Binding the m7GTP cap structure to promote translation	71
	SNRPG	Proto-oncogenic	Glioblastoma	Facilitates the cytosolic translocalization of MYC	72
	DDX31	Proto-oncogenic	Medulloblastoma	Blocking interaction between the E3 ubiquitin ligase and p53	91
Multiple pathway	SOX9	Proto-oncogenic	Medulloblastoma, Glioblastoma	Gene transcription and cell transdifferentiation	28,92,97,98
	SOX2	Proto-oncogenic	Glioblastoma	Maintaining the self-renewal of GSC	80,88
	STAT3	Proto-oncogenic	Glioblastoma	Astrogliogenesis and cell proliferation	84,125,126
	QKI	Proto-oncogenic	Glioblastoma, brain metastasis	Regulator of biogenesis of circular RNAs and exerting functions within EVs	48,108,109
	HuR	Proto-oncogenic	Glioblastoma	mRNA stabilization through m6A modification and 3'-UTR binding	53,68
	HNRNPA2B1	Proto-oncogenic	Glioblastoma	Processing pri-miR and exerting functions within EVs	36,110,111

**Table 2:** The RBP associated treatment entering clinical trials and in preliminary test.

RBP	Therapeutic agent	Drug Types	Cancer	Phase	NCT number	Mechanism	Ref.
WT1	WT1 peptide	Vaccine	Recurrent GBM	II	Not provide	Inducing an antitumor immune response in brain	117
HDAC2	PCI-24781	Inhibitor	Glioblastoma	I	NCT05698524	Enhance sensitivity to temozolomide	121
STAT3	WP1066	Inhibitor	Medulloblastoma, malignant glioma	I	NCT04334863	Target the STAT3 pathway in GBM and activate the immune system	128,129
STAT3	WP1066	Inhibitor	Recurrent Glioblastoma, Metastatic Melanoma	I	NCT01904123	Target the STAT3 pathway in GBM and activate the immune system	128,129
STAT3	WP1066	Inhibitor	Glioblastoma	II	NCT05879250	Target the STAT3 pathway in GBM and activate the immune system	128,129
TERT	GRN163L	Inhibitor	Glioblastoma, Astrocytoma, Oligodendroglioma	II	NCT01836549	Inhibition of telomerase activity	140
LRP1	ANG1005	Inhibitor	Recurrent High-Grade Glioma	II	NCT01967810	Penetrate malignant cells via LRP1 transport system	141
TLR7/8	CDNP-R848	Agonist	Glioblastoma	/	/	Reshapes the immunosuppressive tumor microenvironment to a proinflammatory state	114
TLR9	t-NanoCpG	Agonist	Glioblastoma	/	/	Stimulates the maturation of dendritic cells and production of proinflammatory cytokines	115
EP300	CPI-1612	Inhibitor	Glioblastoma	/	/	SuppressedH3K27ac and the associated transcription complex	122
KDM1A	NCD38	Inhibitor	Glioblastoma	/	/	Increased sensitivity to temozolomide	123
OCT4	CPP-S1	Inhibitor	GSCs	/	/	Inhibitthe palmitoylation of OCT4	124
STAT3	SS-4	Inhibitor	Glioblastoma	/	/	Selectivelyinhibited STAT3 tyrosine (Y)-705 phosphorylation	127
HuR	MS-444	Inhibitor	Glioblastoma	/	/	Inhibition of HuR dimerization	130
NONO	Auranofin	Inhibitor	Glioblastoma	/	/	Disturb the binding and splicing of NONO	131
NPM1	Brequinar, ML390	Inhibitor	Glioblastoma	/	/	Aberrant redistribution of the NPM1	133
METTL3	STM2457	Inhibitor	SHH-medulloblastoma	/	/	Inhibit catalytic function	136
TRIM28	NB237	Inhibitor	GSCs	/	/	Inhibition of GSCs invasiveness and spread	135
SPI1	PLX-4720	Inhibitor	GSCs	/	/	Enhance radiosensitivity through SPI1	35
SPI1	DB2313	Inhibitor	Glioblastoma	/	/	Restore downstream FTO expression	36
CDK9	SNS032, LY2857785, AZD4573, NVP229, JSH15030	Inhibitor	Glioblastoma	/	/	Reduce the cell viability and self-renewal	132
PARP1	PJ-34	Inhibitor	Glioma	/	/	Suppress the brain microvascular endothelial cells migration	134
LIN28B	Inhibitor 1632	Inhibitor	Medulloblastoma	/	/	Inhibit LIN28B-let-7-PBK axis	137
SUMO1	HB007	Inhibitor	Brain tumor	/	/	Selectively degraded SUMO1 through ubiquitination	138
hnRNPK	compound 25	Inhibitor	Glioblastoma	/	/	Disrupt the interaction between hnRNPK and c-myc promoter i-motif	139
PDGFRA	ASO	ASO	Glioblastoma	/	/	ASO targeting lncRNA to block the LINC02283-PDGFRA interaction	145
H3-3A	ASO	ASO	DIPG	/	/	Direct RNase H-mediated knockdown of H3-3A mRNA	146
SRSF1	Decoy oligonucleotides	ASO-like drugs	Glioblastoma	/	/	Inhibit splicing and biological activities of splicing factors	147

## Abbreviations

RBPs: RNA-binding proteins
CNS: Central Nervous System
3'-UTRs: 3' untranslated regions
GBM: glioblastoma
RBDs: RNA-binding domains
RRM: RNA-recognition motif
hnRNP: human heterogeneous nuclear ribonucleoprotein
KH: K-homology domain
RNPs: ribonucleoprotein particles
MLOs: membraneless organelles
ncRNAs: noncoding RNAs
lncRNA: long noncoding RNA
PRC2: polycomb repressive complex 2
H3K27: histone H3 lysine 27
HDAC: histone deacetylase
TMZ: temozolomide
GSCs: glioblastoma stem cells
NMD: nonsense-mediated decay
m6A: N6-methyladenosine
rRNAs: ribosomal RNAs
BBB: blood-brain barrier
EMT: epithelial-mesenchymal transition
IDH: isocitrate dehydrogenase
NSCs: neural stem cells
m5C: 5-Methylcytosine
hm5C: 5-hydroxymethylcytidine
f5C: 5-formyl-cytidine
m1A: N1-methyladenosine
PUSs: pseudouridine synthases
HuR: human antigen R
APA: alternative polyadenylation
SUMO: small ubiquitin-like modifier
LLPS: liquid-liquid phase separation
ECs: endothelial cells
BTB: blood-tumor barrier
EVs: extracellular vesicles
MDSCs: myeloid-derived suppressor cells
RNAi: RNA interference
siRNA: small interfering RNA
123I-MAPi: Iodine-123 Meitner-Auger PARP1 inhibitor
ASO: antisense oligonucleotides
DIPG: diffuse intrinsic pontine gliomas
RIP: RNA immunoprecipitation
TRIBE: targets of RNA-binding proteins identified by editing
CLIP: crosslinking and immunoprecipitation
UV: ultraviolet
HITS-CLIP: high-throughput sequencing CLIP
iCLIP: individual-nucleotide CLIP
eCLIP: enhanced CLIP
PAR-CLIP: photoactivatable ribonucleotide-enhanced CLIP
PEKA: Positionally Enriched K-mer Analysis
RIC: RNA interactome capture
